# A Model of the CA1 Field Rhythms

**DOI:** 10.1523/ENEURO.0192-21.2021

**Published:** 2021-11-08

**Authors:** Ivan Mysin

**Affiliations:** Laboratory of System Organization of Neurons, Institute of Theoretical and Experimental Biophysics of Russian Academy of Sciences, Pushchino 142290, Russian Federation

**Keywords:** gamma rhythm, interneurons, phase coupling, ripple oscillations, theta rhythm, theta–gamma coupling

## Abstract

We propose a model of the main rhythms in the hippocampal CA1 field: theta rhythm; slow, middle, and fast gamma rhythms; and ripple oscillations. We have based this on data obtained from animals behaving freely. We have considered the modes of neuronal discharges and the occurrence of local field potential oscillations in the theta and non-theta states at different inputs from the CA3 field, the medial entorhinal cortex, and the medial septum. In our work, we tried to reproduce the main experimental phenomena about rhythms in the CA1 field: the coupling of neurons to the phase of rhythms, cross-rhythm phase–phase coupling, and phase–amplitude coupling. Using computational experiments, we have proved the hypothesis that the descending phase of the theta rhythm in the CA1 field is formed by the input from the CA3 field via the Shaffer collaterals, and the ascending phase of the theta rhythm is formed by the IPSPs from CCK basket cells. The slow gamma rhythm is coupled to the descending phase of the theta rhythm, since it also depends on the arrival of the signal via the Shaffer collaterals. The middle gamma rhythm is formed by the EPSPs of the principal neurons of the third layer of the entorhinal cortex, corresponds to experimental data. We were able to unite in a single mathematical model several theoretical ideas about the mechanisms of rhythmic processes in the CA1 field of the hippocampus.

## Visual Abstract

**Figure:**
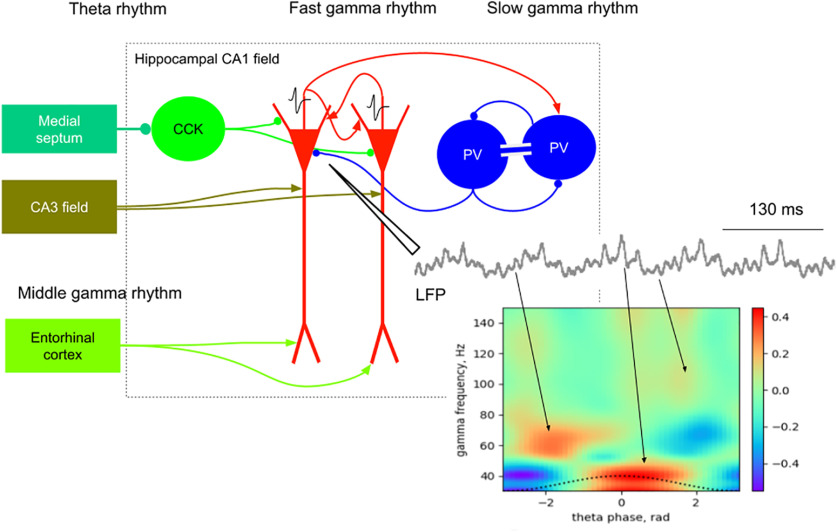

## Significance Statement

The development of models describing a large complex of experimental data are an important aim of theoretical neuroscience. We propose an approach: models of separated aspects can be used as good initial conditions for optimizing a complex model. Using this approach, we have developed a model of rhythms in the CA1 field. We have united theoretical ideas about the formation of theta rhythm, gamma rhythms (slow, medium, and fast), ripple oscillations, and place cells in a single model. The model explains the coupling of neuron firing to the phases of the theta rhythm, the cross-frequency phase–amplitude and phase–phase relationships of gamma rhythms and theta rhythm, the phase precession of place cells. The model reproduces ripple oscillations in the non-theta state.

## Introduction

Despite a century of historical research on brain electrographic rhythms, the mechanisms of rhythm generation, and their role in brain function remain one of the puzzles of modern neuroscience. In this article, we propose a theoretical model of the mechanisms of the generation of basic rhythms in the CA1 field of the hippocampus. We chose the hippocampal CA1 field as the object for modeling for two reasons. First, the hippocampus plays a critical role in attention and memory processes (Vinogradova, 2001; Buzsáki and Moser, 2013). Second, among all hippocampal regions, the CA1 field is the most studied in the context of rhythms (Buzsáki and Wang, 2012; Buzsáki, 2015, 2002). The main goal of our work is to offer a single model of the main rhythms of the hippocampus, as follows: theta (4–12 Hz), slow gamma (30–50 Hz), middle gamma (50–90 Hz), fast gamma (90–150 Hz) rhythms, and ripple oscillations (110–200 Hz).

Based on the literature data, we believe that the main experimental facts that the model of the neural network of the CA1 field should reproduce and explain are as follows. (1) The phase relations of neuronal activity and rhythms: at the peak of the theta cycle, axo-axonal and neurogliaform neurons discharged. Parvalbumin (PV) basket cells fired at the descending phase of the theta cycle. At the trough of the theta cycle, pyramidal, oriens-lacunosum moleculare (OLM), and bistratified neurons have a maximum firing rate. In the ascending phase of the theta cycle, ivy and CCK basket cells are discharged (Somogyi and Klausberger, 2005; Somogyi et al., 2014). (2) Pyramidal neurons during the theta rhythm out of the place field fire infrequently, but the potential varies at the theta frequency (Ylinen et al., 1995; Csicsvari et al., 1998). (3) The model should reproduce the phenomenon of phase precession of place cells relative to the theta rhythm (O’Keefe and Recce, 1993; Skaggs et al., 1996). (4) The model should reproduce the phase coupling of the theta and gamma rhythms. The slow gamma rhythm is most pronounced at the descending phase of the theta rhythm, the middle at the maximum of the theta rhythm, and the fast gamma rhythm at the minimum of the theta rhythm. In addition, there is a constant phase difference between theta and gamma rhythms (Colgin and Moser, 2010; Belluscio et al., 2012; Colgin, 2015, 2016). (5) Antagonistic relations between ripple oscillations and theta rhythm (i.e., the absence of ripple oscillations with a powerful theta rhythm; Buzsáki et al., 1983; Vandecasteele et al., 2014). (6) Parameters of ripple oscillations consist of the form of field potential fluctuations during a ripple event and the activity of neurons of different populations during ripple oscillations (Somogyi et al., 2014; Buzsáki, 2015). (7) The role of external inputs in the CA1 field is explored. Theta rhythm generation requires input from the medial septum (MS; Vinogradova, 1995; Colgin, 2013), slow gamma rhythm and ripple oscillations depend on input via Shaffer collaterals from the CA3 field, and the middle gamma rhythm depends on input via the perforant path from cells of the entorhinal cortex layer 3 [medial entorhinal cortex (MEC); Colgin et al., 2009; Belluscio et al., 2012; Colgin, 2015; Fernández-Ruiz et al., 2017).

Many mathematical models have been proposed in the literature that describe many of the presented experimental observations individually (Wang and Buzsáki, 1996; Saraga et al., 2006; Burgess and O’Keefe, 2011; Börgers et al., 2012; Chance, 2012; Keeley et al., 2017; Mysin et al., 2019). However, there is no model describing all observations in a single model with a single set of parameters. In our work, we tried to combine theoretical ideas about the mechanisms of rhythms, as well as to express some of our ideas about how rhythms are generated.

In experimental studies of the properties of rhythms, more attention is usually paid to the dorsal hippocampus, so our model describes the behavior of the CA1 field network of the dorsal hippocampus.

## Materials and Methods

**Figure 1.**
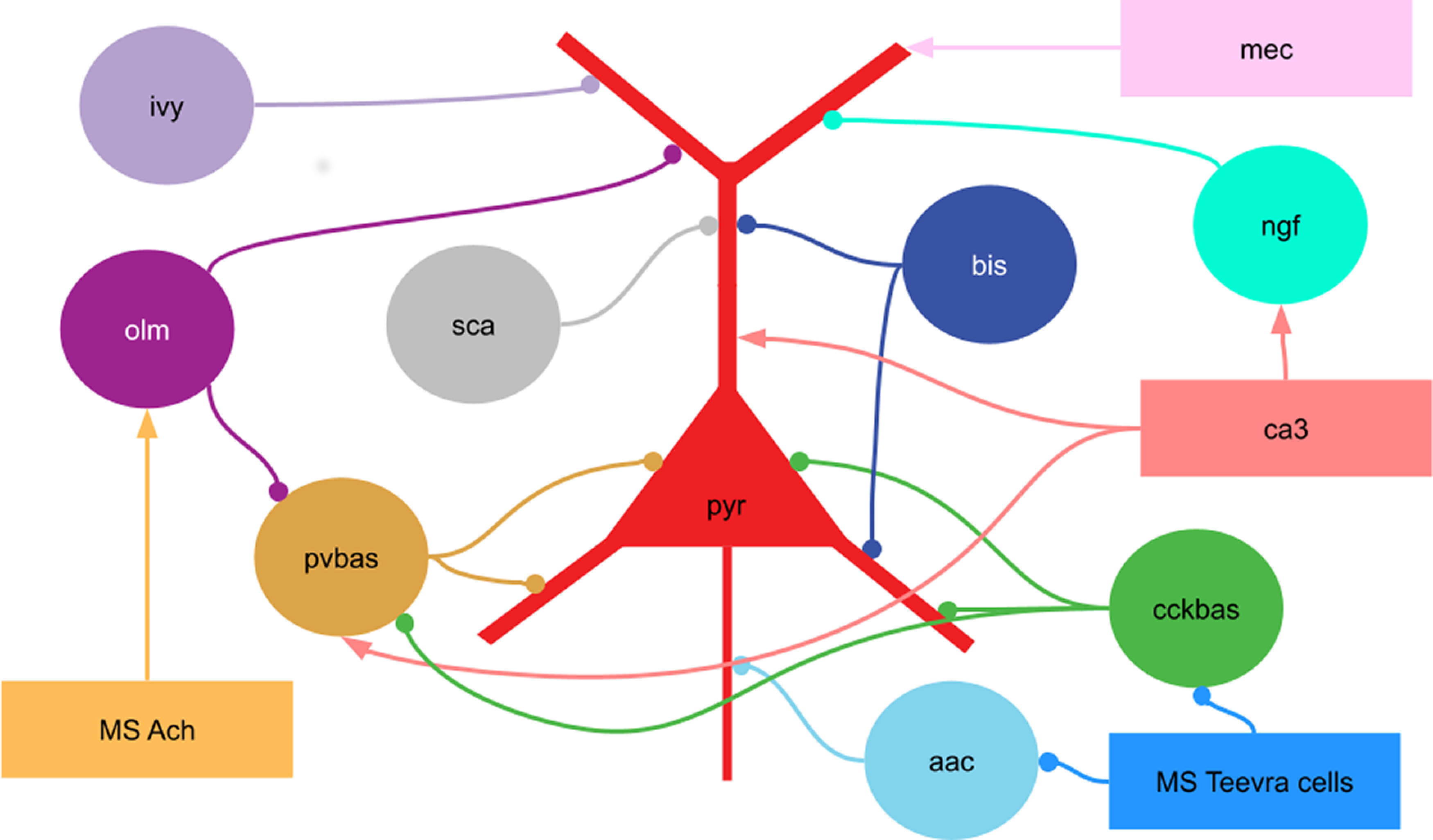
Scheme of the connections in the model. pyr, Pyramidal cells; pvbas, PV basket cells; olm, OLM cells; cckbas, CCK basket cells; ivy, Ivy cells; ngf, neurogliaform cells; bis, bistratified cells, aac, axo-axonic cells; sca, Schaffer collateral-associated cells. Although the interneurons are represented by circles, they were modeled by multicompartment models (see Extended Data 1). The external inputs are shown by rectangles. ca3, input from the CA3 field (spatial and non-spatial); mec, input from the medial entorhinal cortex; MS Ach, input from cholinergic neurons of the medial septum; MS Teevra cells, input from GABAergic Teevra cells of the medial septum. Not all connections are shown in the figure.

Our model included pyramidal neurons (*n* = 9000), as well as eight types of interneurons: CCK (*n* = 160) and PV (*n* = 200) basket cells; axo-axonal cells (*n* = 60); bistratified cells (*n* = 70); OLM cells (*n* = 80); neurogliaform cells (*n* = 130); ivy cells (*n* = 260); and Schaffer collaterals-associated cells (*n* = 40; Fig. 1). To describe the neuron models, we used the Hodgkin–Huxley formalism. All the neurons were multicomponent. Neuron models were taken from previous articles (Cutsuridis and Poirazi, 2015; Bezaire et al., 2016). The connections between the neurons were taken based on the database “hippocampome” (Tecuatl et al., 2021), and GABA_A_ receptors were modeled for inhibitory synapses, nicotine receptors, and AMPARs for excitatory connections. Gap junctions between PV basket cells and between neurogliaform cells were included in the model in accordance with the experimental data (Fukuda and Kosaka, 2000, 2003; Price et al., 2005).

We have simulated external inputs from the CA3 field, the third layer of the entorhinal cortex, and the GABAergic and cholinergic input from the MS. The inputs from the CA3 field were divided into spatially modulated and spatially nonmodulated. The first ones imitated the activity of place cells in the CA3 field, having a peak of discharges in time, these generators showed modulation by theta rhythm (7 Hz) and slow gamma rhythm (35 Hz). Spatially unmodulated generators of the CA3 field imitated the background activity of neurons of this field, showing modulation only by the theta rhythm. Generators simulating the input from the MS with a different phase shift showed similar activity. The MEC generators were modeled as grid cells, with slow periodic activity. They were also modulated by the theta rhythm and the middle gamma rhythm. The mathematical apparatus for describing spike generators and their parameters is described in the supplemental material.

To simulate the non-theta state, we removed the MEC input, spatially unmodulated CA3 generators, and the cholinergic input from the MS from the model. The Teevra cells went into a constant discharge mode (Viney et al., 2013). Spatially modulated generators were modulated by a slow gamma rhythm (35 Hz) and ripple oscillations (170 Hz), and the centers of their discharges converged.

The weights of connections from spatially modulated CA3 generators to pyramidal and PV basket neurons were drawn from a Gaussian distribution. The weights of the connections from the MEC generators to the pyramidal neurons were also distributed using the Gaussian function, but the peak of excitation from MEC was earlier than the peak of excitation from the CA3 field by 500 ms. The place cell first receives input from the MEC and then from the CA3 field (see Fig. 7). PV basket neurons, in turn, inhibited mainly distant pyramidal neurons, the experimental literature provides indirect evidence of such a distribution of connections (Hangya et al., 2009; Udakis et al., 2020). All other connections between neurons were established randomly, the density and other parameters are given in the Extended Data 1. Pairs of PV and neurogliaform neurons for the gap junctions were randomly selected in accordance with their density. The parameters of the gap junctions between the PV neurons did not depend on the cell coordinates.

The local field potential (LFP) has been simulated using linear approximation (Parasuram et al., 2016). The simulations were performed with modified LFPsimpy https://github.com/ivanmysin/LFPsimpy. For parameters, see the Extended Data 1. The data analysis was conducted in a way similar to that for the experimental work. The methods of analysis are also given in the Extended Data 1.

10.1523/10.1523/ENEURO.0192-21.2021.ed1 Extended Data 1 Latex supplement. Download Extended Data 1, ZIP file.

Simulations were performed with an 80 CPU cluster (Intel Cascade Lake) with 160 gigabytes of RAM. It was run under Ubuntu 20.04. The code of the model is available on GitHub (github.com/ivanmysin/CA1_rhythms_model) and also as the Extended Data 2.

10.1523/10.1523/ENEURO.0192-21.2021.ed2 Extended Data 2 CA1 rhythms_model-master. Download Extended Data 2, ZIP file.

## Results

### General characteristics of the model

In our work, we tried to combine many theoretical ideas about how the main rhythms are formed in the CA1 field of the hippocampus. From a practical point of view, the purpose was to optimize the parameters of the model, primarily the weights of connections between populations of neurons, in such a way as to harmonize theoretical ideas about the formation of different rhythms. In the process of optimizing the parameters of the model, the following two tasks can be distinguished: the explanation of the shape of the field potential and the dynamics of the discharges of neurons during rhythmic processes.

According to modern concepts, the field potential is formed by currents in the extracellular environment, which are produced by currents through the membranes of pyramidal neurons. The currents through the membranes of pyramidal neurons are summed up because of the ordered structure of the processes of pyramidal neurons (Buzsáki et al., 2012; Parasuram et al., 2016). Based on the analysis of LFP signals by the current source density method, it is possible to identify which layers areinvolved in the generation of each rhythm. When constructing our model, we optimized the parameters so that the inputs from the CA1 interneurons and external inputs create the corresponding currents and provide depolarization or hyperpolarization of the corresponding compartments of the pyramidal neurons. For example, with the slow gamma rhythm, the maximum currents occur between the stratum radiatum and the stratum oriens, which corresponds to the excitation of pyramidal neurons by Shaffer collaterals and the inhibition by PV basket cells, so the weights of connections from these neurons to the pyramidal neurons in our model are large.

In the CA1 field, almost all neural populations are modulated by rhythms. The problem of forming the dynamics of neuronal activity observed in the experiment was solved by optimizing the parameters of connections from external inputs and between neurons in such a way that they formed the experimentally observed dynamics relative to each other. For example, during the theta rhythm, PV and CCK basket neurons are discharging in opposite phases, descending and ascending, respectively. In our model, we established large weights of connections between them. Thus, antiphase relationships are formed between these neural populations.

**Figure 2.**
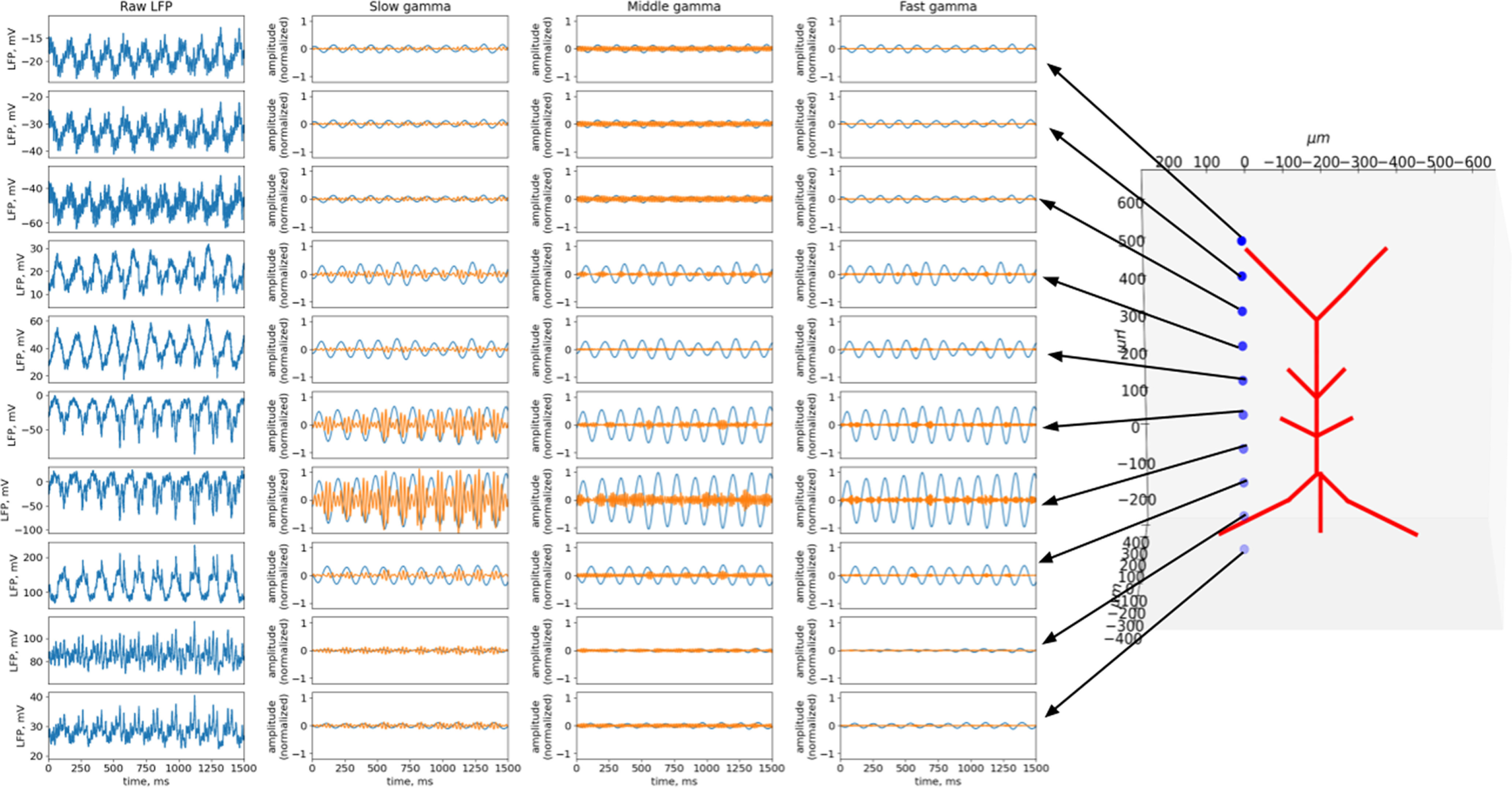
Raw LFP and oscillation bands dynamics across layers in the theta state. The left column of the plots shows a simulated raw LFP signal. Note: plots are displayed on different scales. On the right, the signals in the theta and gamma bands are normalized to the power of the raw LFP. The morphology of the pyramidal neuron is shown on the right. The blue dots indicate the sites where the LFP was calculated.

**Figure 3.**
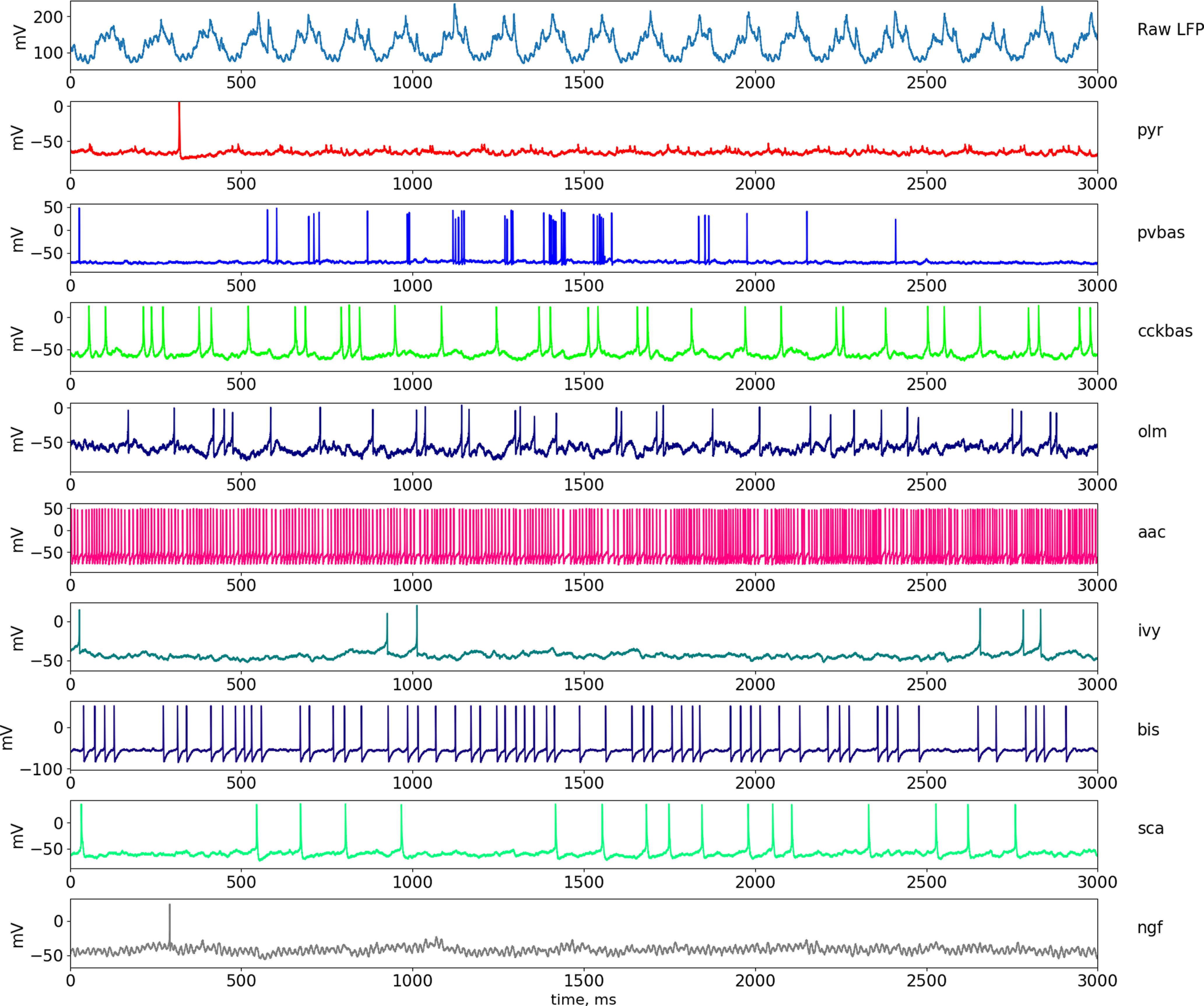
Dynamics of LFP and neuronal activity in the theta state. The top part of the panel shows the simulated LFP in the pyramid layer. Examples of the dynamics of intracellular potentials on the soma of neurons are also presented below. Abbreviations are the same as in Figure 1.

**Figure 4.**
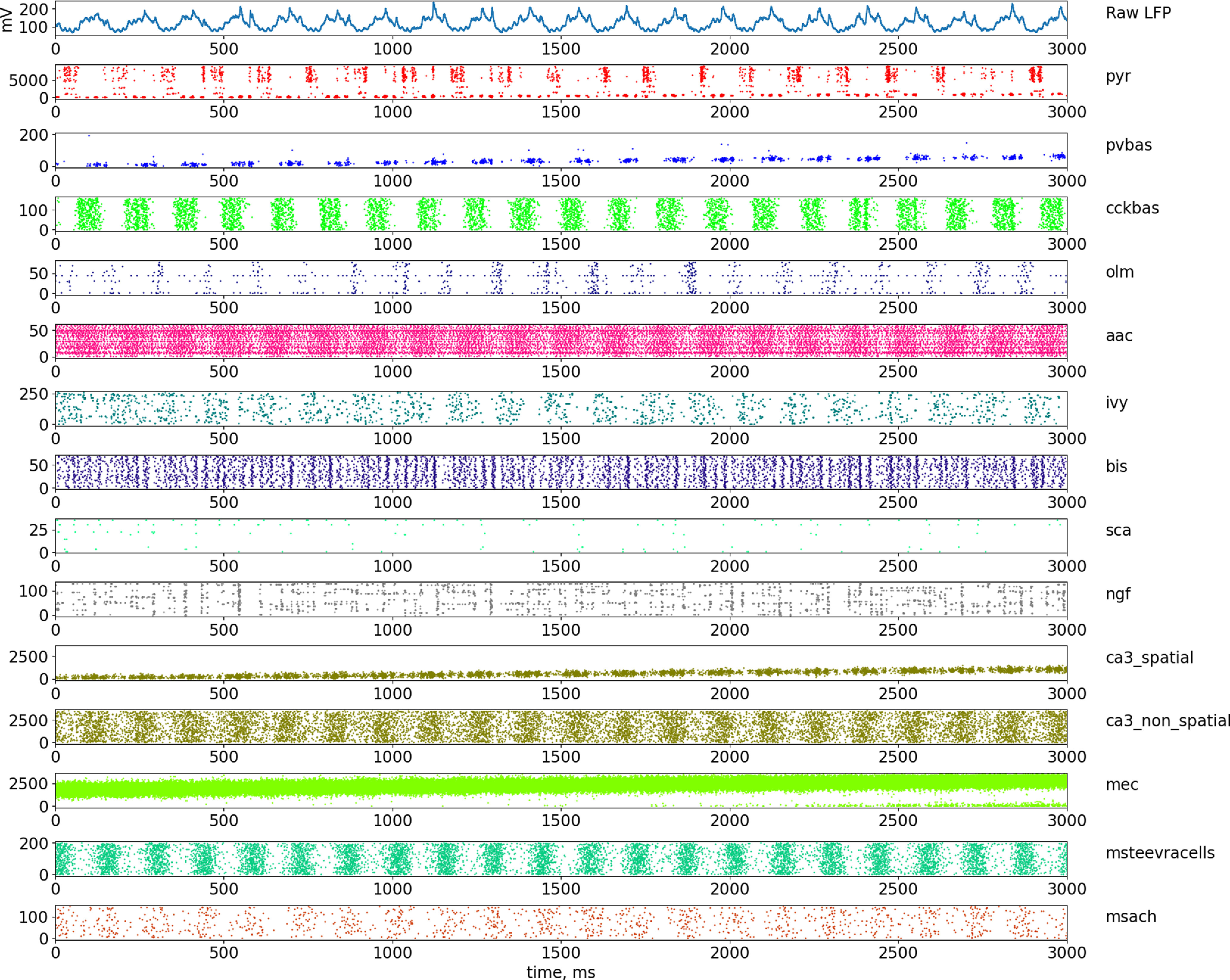
Dynamics of LFP and neuronal firing in the theta state. The top part of the panel shows the simulated LFP in the pyramid layer. The lowest series of plots show raster graphs of the discharges of neurons. Abbreviations are the same as in Figure 1.

Figures 2, 3, and 4 show the results for the theta state (see Fig. 8 for the results of the non-theta state). These results correspond to the experimental data listed in the introduction. In the following sections, we describe in detail how we obtained these results.

### Model of theta rhythm

**Figure 5.**
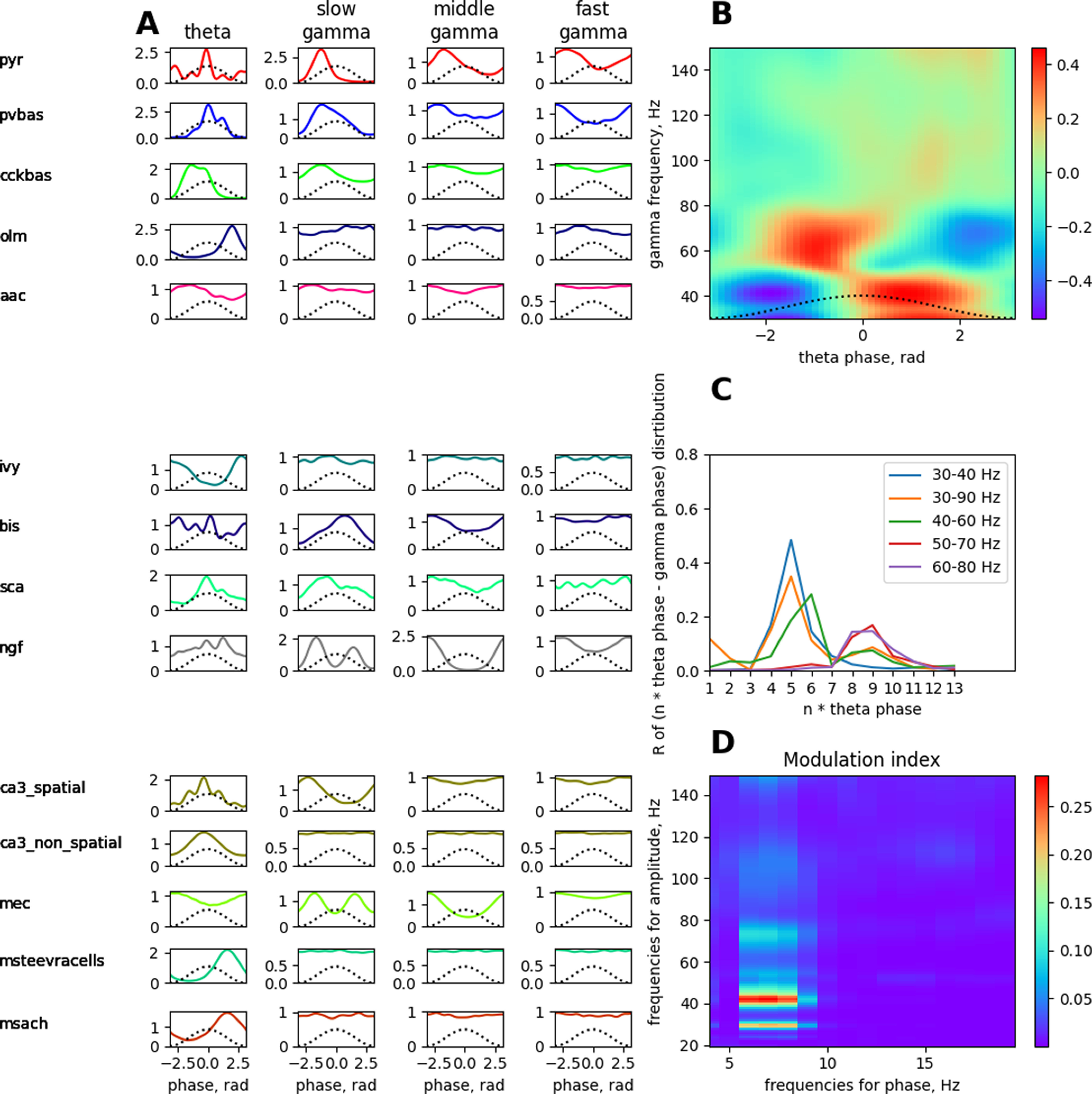
Phase analysis of the dynamics of neurons relative to LFP rhythms and cross-rhythm relations in the theta state. A simulated LFP signal from the pyramid layer was used to construct all the plots. ***A***, Distribution of spikes by rhythm phases. ***B***, Amplitude–phase coupling of gamma rhythms and theta rhythms. ***C***, Phase–phase coupling of theta and gamma rhythms in n:m-test. ***D***, The modulation index of the amplitude from the range of the gamma rhythm by the phase of the theta rhythm. Abbreviations are the same as in Figure 1.

The theoretical ideas about the mechanisms of theta rhythm suggested in this article are based on our previous work (Mysin et al., 2019). In our previous study, we showed that input from the CA3 field via Shaffer collaterals plays a key role in theta rhythm generation in the CA1 field. This hypothesis explains the biological plausibility of the activity of pyramidal and PV basket neurons during the theta cycle. In this work, other types of interneurons have added to our model, in particular CCK basket cells. We believe that CCK basket neurons are also important for theta rhythm generation. The discharges of these cells occur in the ascending phase of the theta rhythm in the pyramidal layer of the CA1 field (Fig. 5; Somogyi and Klausberger, 2005), which coincides with the hyperpolarization of the soma of pyramidal neurons. In addition, CCK basket neurons receive very powerful input from the medial septum (Joshi et al., 2017). Thus, in this model, the theta cycle is formed by two mechanisms: depolarization of the perisomatic zone of pyramidal neurons by the input from Shaffer collaterals in the descending phase of the theta rhythm and hyperpolarization of the soma of pyramidal neurons by the inputs from CCK basket neurons. The rhythmic activity of Shaffer collaterals is embedded in the model. The rhythmicity of the CCK basket neurons occurs because of the presence of two inputs from the medial septum—cholinergic and GABAergic (Joshi et al., 2017). The cholinergic input has a muscarinic nature and is modeled by the presence of a tonic excitatory current in the CCK basket neurons. GABAergic input is provided by rhythmic spike generators that mimic the input from the Teevra cells of the medial septum (Joshi et al., 2017). Because of these two mechanisms, CCK basket cells are introduced into a stable rhythmic mode.

**Figure 6.**
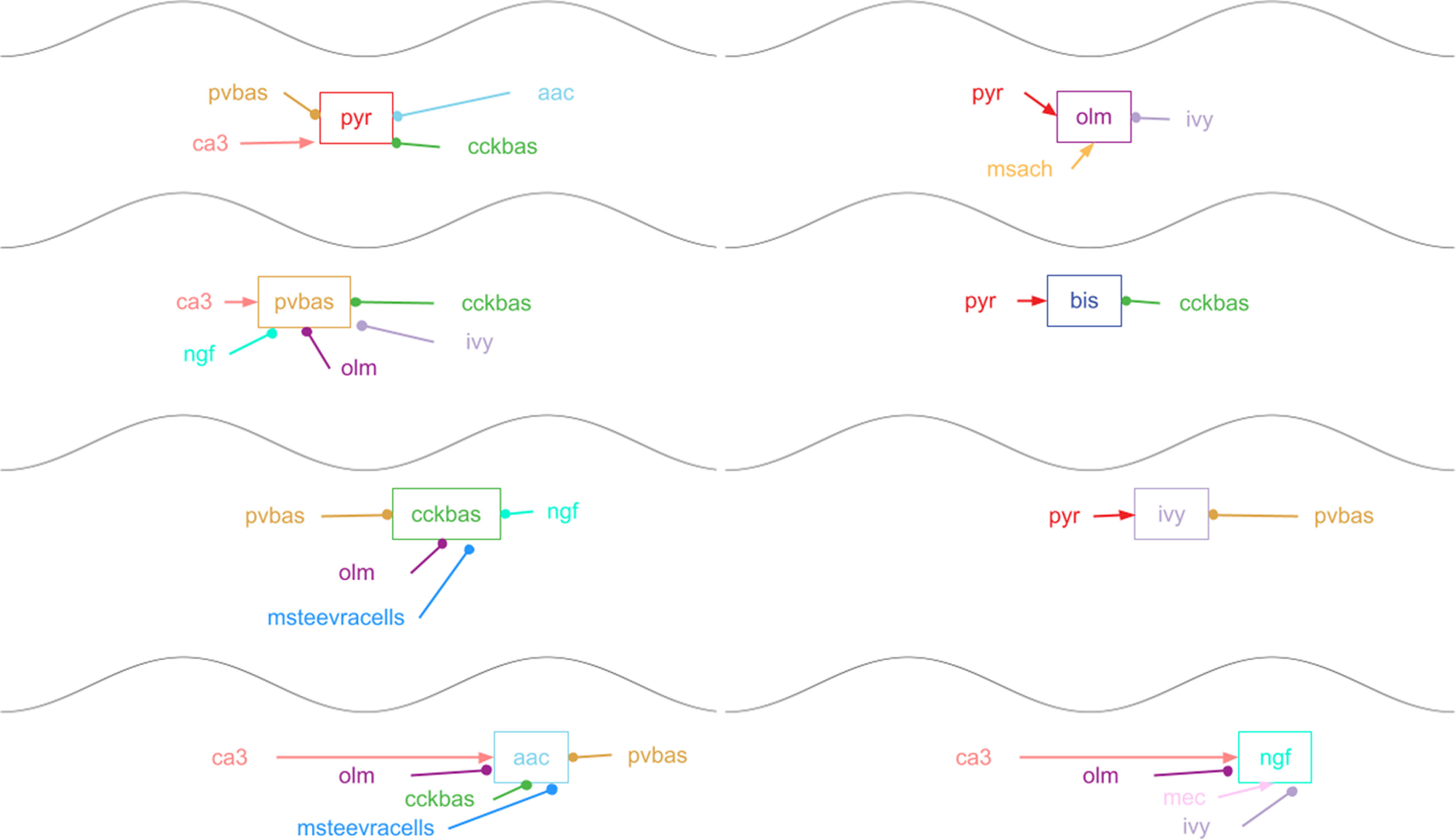
The scheme of сoupling between neuronal population firing and the theta rhythm phase. Abbreviations are the same as in Figure 1.

The role of interneurons, except for the CCK basket neurons, in the generation of theta rhythm, is currently unclear. Available experimental data on PV basket and OLM neurons indicate that these cells do not play a critical role in the generation of theta rhythm in the CA1 field (Royer et al., 2012). We found no experimental data on the functions of the other neurons. In our model, we optimized the parameters of connections from other interneurons, except for the CCK basket neurons, so that their inputs to the pyramidal neurons do not destroy the theta rhythm mechanism described above. The connections between the populations of interneurons were optimized so that they formed the correct coupling of the discharges to the theta rhythm phase (Figs. 5*A*, 6).

The coupling of neuronal discharges is formed by external inputs and connections inside the CA1 field. For example, PV basket neurons fire at the descending phase of the theta rhythm, because they are excited by the Shaffer collaterals. Neurogliaform neurons inhibit them at the peak of the theta rhythm, and OLM interneurons at the minimum. In turn, neurogliaform and OLM neurons are controlled by external inputs and other local connections. The complete scheme of the formation of all phase relations is shown in Figure 6.

The plots in Figure 5*B–D* show the coupling of theta and gamma rhythms. The maximum power of the slow gamma rhythm is located in the descending phase of the theta rhythm, and the middle gamma rhythm is most pronounced in the ascending phase of the theta rhythm near the peak of Figure 5*B*. This corresponds to the data (Belluscio et al., 2012). The graph in Figure 5*C* shows the phase–phase coupling (n:m test) of theta and gamma rhythms. The peaks show that when the theta band (4–12 Hz) of the LFP signal is “accelerated” by five and nine times, it becomes coherent with gamma oscillations (the ranges are signed in the figure; Belluscio et al., 2012). Figure 5*D* demonstrates the strength of the dependence of amplitudes in the gamma band from phases in the theta band. The theta rhythm modulates the slow and middle gamma rhythms most strongly (Colgin et al., 2009).

**Figure 7.**
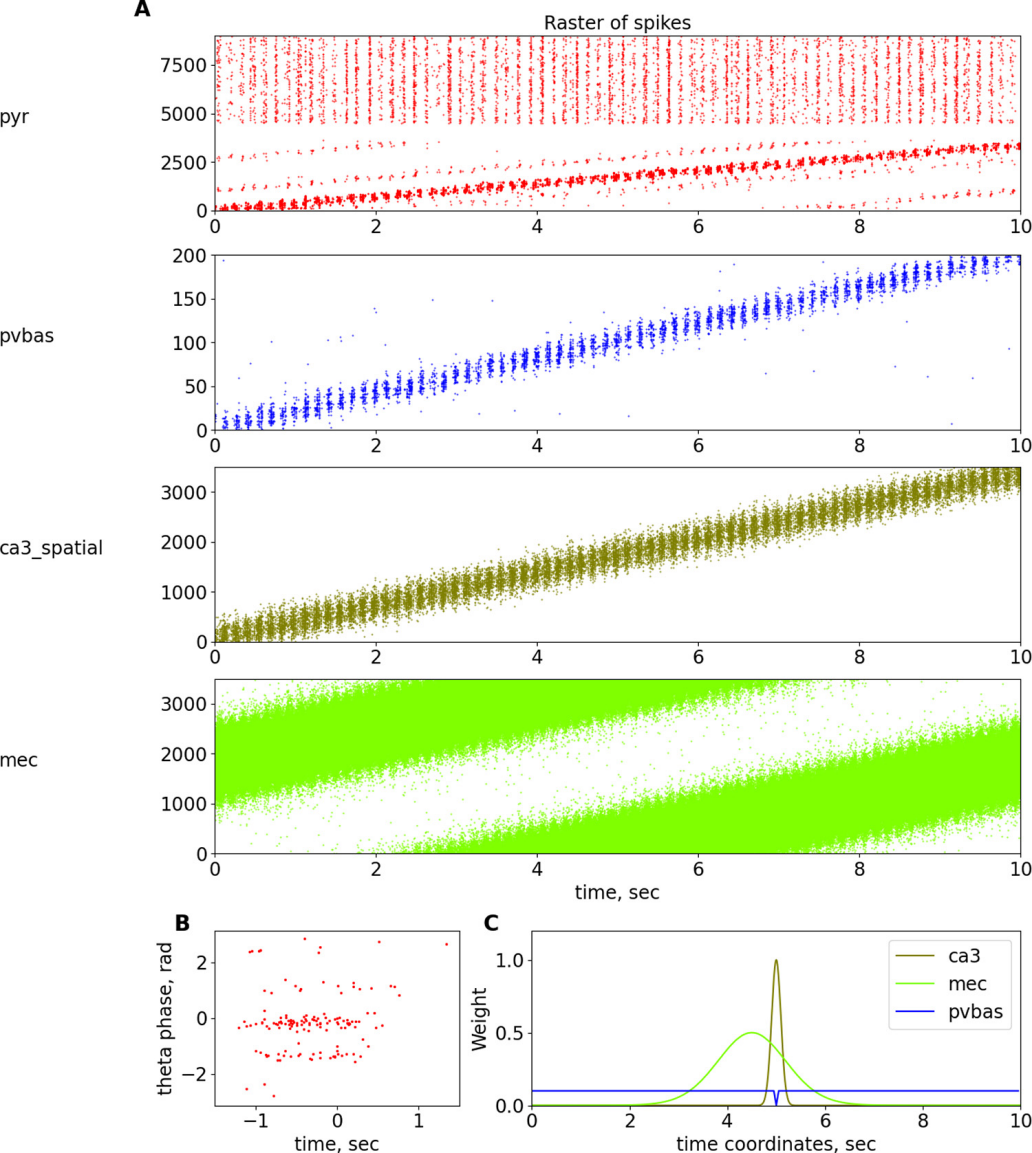
Place cells. ***A***, Raster plots of spatially modulated inputs and model neurons. ***B***, Phase precession of pyramidal neurons. ***C***, Dependence of weights of connections from ca3_spatial, mec, and pvbas to the pyramidal cells from their coordinates. For details, see Table 22 in Extended Data 1. Abbreviations are the same as in Figure 1.

It should be noted that the distribution of firing of pyramidal neurons in the theta rhythm phase is bimodal in our model (Fig. 5*A*). Pyramidal neurons outside the place field are essentially discharged at the minimum of the theta rhythm. However, inside the place field, the inhibition of PV basket cells is less (Fig. 7*C*), so the pyramidal neurons begin to discharge at the descending phase of the theta rhythm. In our model, the ratio of spatially modulated and unmodulated pyramidal neurons is 1:1, which creates two pronounced peaks on the theta rhythm phase distribution plot. The discharges of the place cells in the descending phase of the theta rhythm in our model play a phase role in the formation of a slow gamma rhythm, which we will discuss below. We could achieve a single dominant peak at the theta rhythm minimum by increasing the number of spatially unmodulated pyramidal neurons, but this would increase the computational complexity of the model.

There is another problem that we could not solve within the framework of the accepted hypotheses. It is the high firing rate of axo-axonal cells (Figs. 3, 4). Axo-axonal neurons control the activity of pyramidal neurons. If the activity of axo-axonal cells is lowered, then this will affect other characteristics of the model through pyramidal neurons. There are only 60 U of axo-axonal cells in the model. We believe that this problem is not important for the mechanisms of rhythms. In a larger-scale model with an increased number of neurons of all types, it is possible to reduce the firing rate of axo-axonal cells with an increase in the density of connections from them.

### Place cells and phase precession

In our work, we focused on the mechanisms of rhythms, but we could not avoid the topic of place cells, because the phenomenon of place cells is closely related to the mechanisms of rhythm generation. In our model, we will not consider the processes of learning, place cell formation, and remapping. The place cells in our model are present only to the extent that we believe is necessary to investigate the mechanisms of the rhythms. The activity of pyramidal neurons as place cells is inherited from inputs from the CA3 field and the entorhinal cortex (Fig. 7). It is shown that pyramidal neurons are modulated by slow gamma rhythm, and when they are discharged the animal is inside the place field of these neurons (Senior et al., 2008). This will be discussed in more detail later in the sections on gamma rhythm.

Another important physiological phenomenon that links rhythmic field activity and cell discharges is phase precession (O’Keefe and Recce, 1993; Burgess and O’Keefe, 2011). A large number of theoretical explanations of the phase precession phenomenon have been proposed, although most hypotheses are reduced to the following two theoretical ideas: the interference of two-oscillator inputs and the asymmetry of one exciting input (Burgess and O’Keefe, 2011). In our work, we will adhere to the hypothesis based on the interference of two inputs, from the CA3 field and the entorhinal cortex (Chance, 2012; Fernández-Ruiz et al., 2017). In our model, we optimized the parameters, focusing primarily on the data of the article (Fernández-Ruiz et al., 2017), because we believe this hypothesis is the most reasonable. In our model, the input from the entorhinal cortex comes earlier than the input from the Shaffer collaterals, which ensures the beginning of the discharges of the place cells first from one input and then from another (Fig. 7), which ultimately allows us to reproduce the dependence of the theta rhythm phase on the position of the animal for the discharges of the place cells. It should be noted that in our model, the phase precession is not as pronounced as in the special models devoted to this phenomenon. This is because of the fact that our model contains additional elements, in particular CCK interneurons. The presence of many interneurons leads to the fact that pyramidal neurons receive more rhythmic inputs, in contrast to many other models. We could not optimize the parameters of our model for a better description of the phase precession without reducing the quality of the description of the properties of rhythms. We believe that an experimental study of the role of different groups of interneurons in the formation of place cells should provide the necessary data to improve a model of phase precession. We did not introduce additional hypotheses about the spatial modulation of different populations of interneurons, so as not to complicate the model.

### Model of slow gamma rhythm

Currently, it has been proved that PV basket neurons play a critical role in the generation of the slow gamma rhythm. There are two theoretical schemes to explain the mechanisms of their synchronization (Colgin and Moser, 2010; Buzsáki and Wang, 2012). The first mechanism involves the synchronization of the PV basket neurons with each other because of inhibitory synapses and gap junctions (Wang and Buzsáki, 1996; Saraga et al., 2006). The second mechanism involves the synchronization of pyramidal neurons and the excitation from them of the PV basket neurons, which in turn give inhibitory feedback to the pyramidal neurons (Börgers et al., 2012; Keeley et al., 2017). The second mechanism has the most experimental confirmation, but it does not contradict the first one. In our model, we included both mechanisms. We included gap junctions between PV basket neurons. Also, the synapses between pyramidal and PV basket neurons are optimized in such a way that inhibitory feedback between them was active.

Many studies have shown that slow gamma occurs in the descending phase of the theta rhythm in the pyramidal layer, and it is a consequence of the arrival of the signal from the Shaffer collaterals (Colgin et al., 2009; Belluscio et al., 2012; Colgin, 2015; Fernández-Ruiz et al., 2017). As we discussed in the section on the mechanisms of the theta rhythm, we believe that the descending phase of the theta rhythm is provided by the Shaffer collaterals input. The excitation from the Shaffer collaterals leads to the excitation of a part of the pyramidal neurons, from which the PV basket neurons are excited. Further, the PV neurons inhibit the pyramidal neurons, and, as a result, negative feedback is formed, which in the field potential manifests itself as a slow gamma rhythm. The coupling of theta and gamma rhythms consists of a single mechanism: the descending phase of the theta rhythm indicates the arrival of a signal via the Shaffer collaterals (Fig. 4).

The presence of gap junctions between the PV basket neurons and strong connections between the pyramidal and PV basket neurons creates the possibility of forming gamma oscillations in the CA1 field. The pyramidal neurons of the CA3 field also have modulation at the gamma frequency, the rhythmic input by the Shaffer collaterals resonates with the endogenous mechanisms of gamma rhythm generation in the CA1 field, and, as a result, we observe high coherence at the gamma frequency between the signals registered in the CA3 and CA1 fields (Colgin, 2015; Fernández-Ruiz et al., 2017). In our model, only the spatially modulated generators that mimic the place cells in the CA3 field showed modulation by the slow gamma rhythm. This allows us to explain the experimental fact that the activity of pyramidal neurons inside their place field is more strongly modulated by a slow gamma rhythm than outside the field of place (Senior et al., 2008).

The coupling parameters of the other interneurons (except for the PV basket interneurons) were not optimized for simulating the slow gamma rhythm (Fig. 5*A*). We did this for two reasons. On the one hand, the literature does not show the participation of other groups of interneurons in the generation of a slow gamma rhythm. On the other hand, the slow gamma rhythm is most powerful in the descending phase of the theta rhythm, at this phase of the theta rhythm, only the PV basket neurons have a peak of activity, the other interneurons are discharged little, and, as a result, their action potentials play a small role in the dynamics of the network.

### Model of middle gamma rhythm

Currently, theoretical models of the middle gamma rhythm are not proposed. However, the authors of the experimental articles agree that the middle gamma rhythm in the CA1 field is a consequence of the arrival of a signal from the entorhinal cortex via the perforant path, and it is also well established that the middle gamma rhythm is associated with the theta rhythm (Colgin et al., 2009; Belluscio et al., 2012; Colgin, 2015; Fernández-Ruiz et al., 2017). In our model, the input from the entorhinal cortex was emitted by artificial spike generators that were modulated at the frequency of the middle gamma rhythm (63 Hz). Thus, in our model, the mechanism of the middle gamma rhythm consists of induction by the entorhinal cortex, which corresponds to the experimental data (Figs. 2, 3, 5).

### Model of fast gamma rhythm

No theoretical models of the fast gamma rhythm have been proposed in the literature. Experimental data on this rhythmic process are scant. The maximum power of the fast gamma rhythm is observed in the pyramidal layer of the CA1 field near the minimum of the theta rhythm (Fernández-Ruiz et al., 2017), during the maximum firing of pyramidal neurons (Somogyi et al., 2014; Figs. 2, 3, 5). These two facts allow us to hypothesize that the fast gamma rhythm is formed because of the generation of action potentials by pyramidal neurons. In our model, the mechanism of the fast gamma rhythm is based on this hypothesis.

### Model of ripples oscillations

Several models of ripple oscillations have been proposed in the literature (Stacey et al., 2009; Taxidis et al., 2012, 2013; Jahnke et al., 2015; Omura et al., 2015; Malerba et al., 2016), describing the signal form of the field potential during the generation of a ripple event, the dynamics of neuron activity during a ripple event, as well as the phase relationship between neuron activity and field potential (Klausberger et al., 2005; Varga et al., 2012; Buzsáki, 2015). Experimental data indicate that the mechanism for generating ripple oscillations is the rapid synchronization of pyramidal neurons because of excitatory connections between themselves. Such activity is more likely to occur in the dentate gyrus or the CA3 field because there are very strong collateral connections between the principal neurons, and this activity is then projected into the CA1 field (Nakashiba et al., 2009). In our model, to simulate the transition of the hippocampal network to the non-theta state, we changed the parameters of external inputs. The input from the Shaffer collaterals became fast, and the input from the Teevra cells became constant (Joshi et al., 2017). Constant input from the Teevra cells led to constant inhibition of the CCK basket and axo-axonal neurons, which led to a decrease in the perisomatic inhibition of pyramidal neurons. Reduced perisomatic inhibition makes it possible for pyramidal neurons to discharge at a higher frequency.

**Figure 8.**
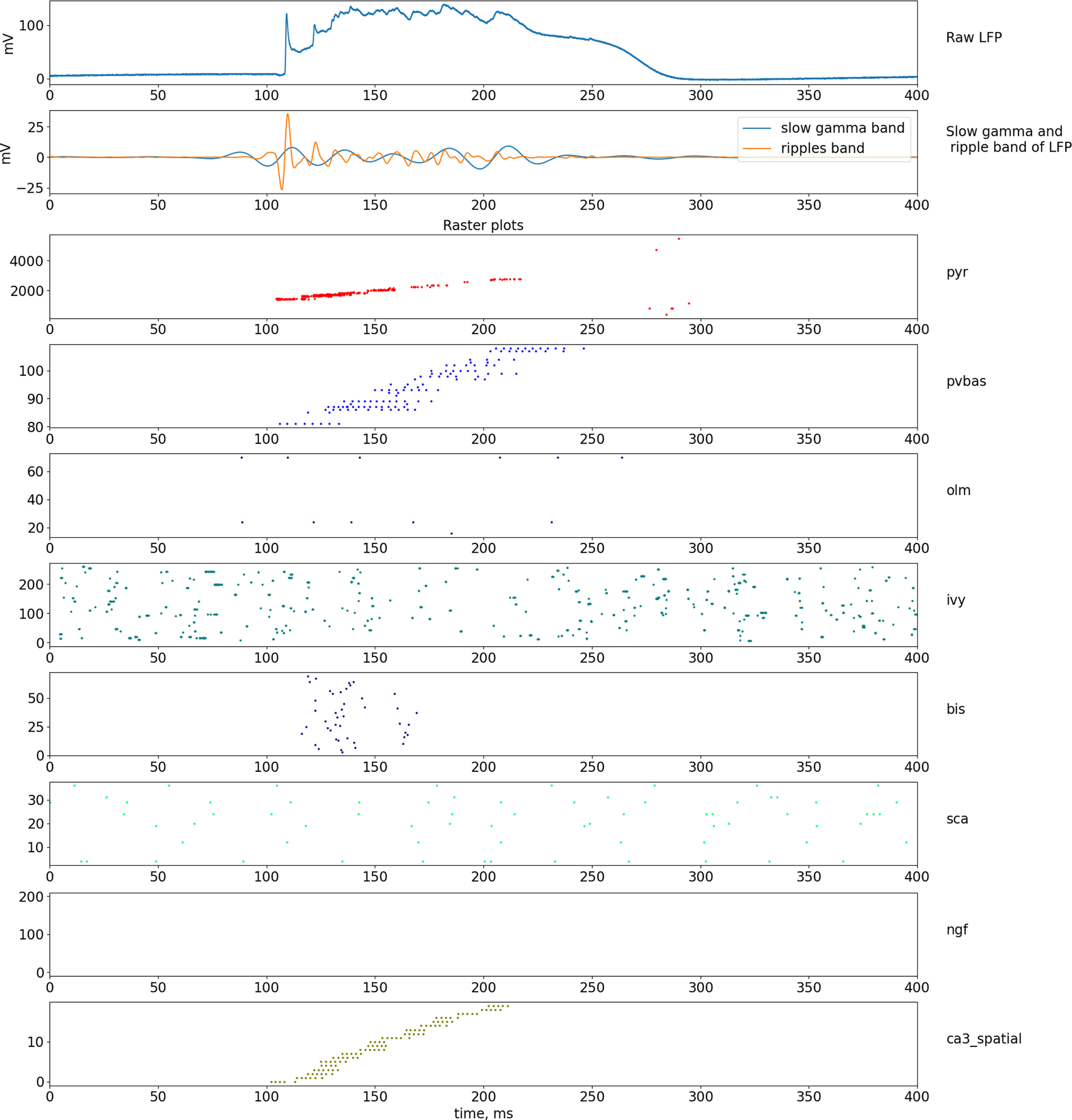
Dynamics of LFP and neuronal activity in the non-theta state. The top part of the plot shows the simulated LFP in the pyramid layer, and the signals in the frequency bands of the slow gamma rhythm and ripple oscillations are shown below. The lowest series of plots shows raster graphs of the spikes. Abbreviations are the same as in Figure 1.

In our ripple model, the event is completely formed by the input from the CA3 field. To simulate a ripple event, we reduced the discharge time of the CA3 generators by 40 times, while the discharge sequence time was ∼100 ms (Fig. 8), which corresponds to the time parameters of ripple events (Buzsáki, 2015). The generators simulating the input from the CA3 field were modulated by the frequency of a slow gamma rhythm (35 Hz) and ripple oscillations (170 Hz), which corresponds to experimental data (Carr et al., 2012).

## Discussion

### General notes

In our work, we first proposed a model of the neural network of the hippocampal CA1 field, which reproduces two main rhythmic states: theta and non-theta state, depending on the parameters of external inputs. Our model describes the role of pyramidal neurons and eight types of interneurons in the generation of rhythmic processes of the CA1 field. One of our main goals was to reproduce and explain the dynamics of field potential fluctuations recorded *in vivo*. Therefore, in our work, we tried to take into account in detail the external inputs to the CA1 field from the medial septum, the CA3 field, and the entorhinal cortex. Unlike many theoretical works, we did not try to get a rhythmic mode inside our model, almost all rhythms in our model are induced from external inputs. On the one hand, this corresponds to the experimental data obtained *in vivo*. On the other hand, the question of the emergence of synchronous modes in neural networks is studied in detail in theoretical works. The occurrence of theta synchronous activity in the MS is considered in several articles (Ujfalussy and Kiss, 2006; Mysin et al., 2015). The occurrence of slow gamma rhythm and ripple oscillations has also been considered in numerous studies (Wang and Buzsáki, 1996; Saraga et al., 2006; Börgers et al., 2012; Taxidis et al., 2012, 2013; Jahnke et al., 2015; Omura et al., 2015; Malerba et al., 2016; Keeley et al., 2017). We tried to combine already known theoretical concepts in a single model and obtain a model that describes the largest possible set of experimental data on rhythms in the CA1 field. As a result, we were able to make a single model that describes all the experimental data listed in the Introduction.

There is an important question. How possible is it to find other combinations of parameters and a different structure of the model that would provide the same degree of agreement with the experimental data? Another combination of parameters most likely exists, but it is a very difficult task to find it. If we take other initial conditions and another optimization method, then perhaps the result will be better. However, it is impossible to iterate over all the variants of the initial conditions. We chose a reasonable strategy for determining the initial conditions. Our strategy has led to good results.

### Theta rhythm model

Our contribution to the proposed general model was to develop a theta rhythm model. In this section, we want to discuss in detail the proposed mechanism of the theta rhythm. Unlike other rhythms, we completely ignored the theta rhythm models proposed by other authors (Orbán et al., 2006; Neymotin et al., 2013; Ferguson et al., 2015; Bezaire et al., 2016). This is because the proposed theta rhythm models contradict a lot of important experimental data. In particular, many of the models explain the occurrence of theta rhythm as a synchronization process that occurs locally in the hippocampus. However, the overwhelming amount of experimental data suggests that synchronous theta activity of neurons occurs in the medial septum, then because of GABAergic projections, it is transferred to the hippocampus (Vinogradova, 1995; Vertes and Kocsis, 1997; Buzsáki, 2002; Colgin, 2013). Theta frequency oscillations can occur in the hippocampus *in vitro* experiments under the influence of strong excitatory agents, such as carbacholine (Williams and Kauer, 1997; Traub et al., 2004; Kazmierska and Konopacki, 2013). However, this activity is very different from the theta rhythm, which is observed in animals in free behavior. The main difference is the number of simultaneously active pyramidal neurons. When generating a theta rhythm in animals in free behavior, only ∼1% of CA1 pyramidal neurons are discharged in a single theta cycle (Ylinen et al., 1995), similar experimental estimates exist for the CA3 field and the dentate gyrus (Soltesz et al., 1993; Soltesz and Deschênes, 1993). In models based on the idea of synchronization within the hippocampus, the number of simultaneously active pyramidal neurons is very large and close to 100%, since synchronization occurs because of excitatory connections between the principal neurons.

The main ideas about the mechanism of theta rhythm generation are based on the results of our previous article, but we have introduced additions to our theoretical scheme, which should be discussed in detail. One of the main results of our previous article was the idea of Shaffer collaterals as the main input forming the descending phase of the theta rhythm in the CA1 field. Subsequent experimental research using optogenetics methods showed our correctness. When the PV basket neurons were stimulated in the CA3 field, the theta rhythm power in the CA1 field decreased (López-Madrona et al., 2020). Our prediction about the role of the perforant path in theta rhythm generation, made in our previous work, was also confirmed. The perforant path input does not play a role in theta rhythm generation, as confirmed by experiments with signal suppression from the entorhinal cortex (López-Madrona et al., 2020), although the results of the study by López-Madrona et al. (2020) directly contradict the results of previous studies (Middleton and McHugh, 2016). We believe that the difference lies in the methods of research. The article used a genetic modification of CA3 pyramidal neurons (Middleton and McHugh, 2016), while the article (López-Madrona et al., 2020) used optogenetic methods, the effect was acute, without adaptive changes. We believe that the data obtained with optogenetics is more reliable.

A significant completion compared with our previous work was the introduction of CCK basket neurons. No direct data are indicating the important role of these interneurons in the generation of theta rhythm, but there are many indirect ones. This group of neurons receives dense input from the medial septum (Joshi et al., 2017). The discharges of this group of neurons occur in the ascending phase of the theta rhythm (i.e., during the period of hyperpolarization of the soma of pyramidal neurons; Klausberger et al., 2005; Somogyi et al., 2014), from which it is very logical to assume that they provide this hyperpolarization.

The literature presents opposite data on the role of NMDA receptors in theta rhythm. Some authors suggest a large contribution of NMDA receptors to theta rhythm, especially in the blockade of muscarinic receptors (Soltesz and Deschênes, 1993; Buzsáki, 2002). Another study has shown no effect of the NMDA receptor blocker on the theta rhythm power of the injected drug in the hippocampus (Gu et al., 2017). Based on these data, we have not introduced NMDA receptors, so as not to complicate the model.

We have not introduced short-term plasticity to the model. Short-term plasticity affects the patterns of neuronal discharges, provided that the input parameters change over time. The period of the slowest rhythm in our model, the theta rhythm, is 140 ms, which corresponds to the characteristic times of short-term plasticity in tens and hundreds of milliseconds (Fioravante and Regehr, 2011). We consider the flow of incoming firing patterns with constant characteristics. In this case, the network will come to equilibrium and the weights of the synapses will stop changing. We can say that we have considered the situation of an established equilibrium.

To reproduce the experimental results in our model, the phase relations of the external inputs are extremely important. The phase difference between the inputs from the CA3 field and the MEC is known from experimental data (Mizuseki et al., 2009). It is also known that the inputs from the Teevra cells coupling to the minimum of the theta cycle (Joshi et al., 2017). The phase relations between the inputs from the CA3 field and the Teevra cells are very important for our model since they allow us to specify the excitation and inhibition of the perisomatic zone of pyramidal neurons. There is also a cholinergic input to OLM neurons via nicotine receptors (Haam et al., 2018). Although there are currently no reliable data on the phase relationship of cholinergic neurons and theta rhythm, we assumed that it falls on the minimum of theta rhythm. This hypothesis allows us to strengthen the binding of OLM neurons to the minimum of the theta cycle, this input in our model has an auxiliary value.

### Key assumptions and predictions about the slow, middle, and fast gamma rhythms, ripple oscillations, and phase precession

Models of slow gamma rhythm, ripple oscillations, and phase precession are taken almost entirely from the cited works, so we can only refer to the originals to discuss these aspects. We will simply list the most important assumptions and consequences without a detailed discussion, as follows. (1) The pyramidal neurons that make up the active neural ensemble (currently active place cells) form a slow gamma rhythm by receiving more excitation from the CA3 field and the entorhinal cortex (Senior et al., 2008; Colgin et al., 2009; Fernández-Ruiz et al., 2017). (2) Spatial modulation of the PV basket neurons is formed by input from CA3 and local pyramidal neurons (Royer et al., 2012). (3) The phase precession is formed by the input from the entorhinal cortex and the CA3 field with a phase and time shift (O’Keefe and Burgess, 2005; Chance, 2012; Fernández-Ruiz et al., 2017).

In our model, the middle gamma rhythm is formed by simple induction from the entorhinal cortex. The maximum power of the middle gamma rhythm is observed in the lacunosum moleculare layer (Fernández-Ruiz et al., 2017). At the moment of arrival of the signal via the perforant path, a current sink is observed in the lacunosum molecular layer, which indicates the predominance of excitation over inhibition (Mizuseki et al., 2009). These two facts support a hypothesis that the middle gamma rhythm is formed by EPSPs from signals coming along the perforant path. In this process, it is possible to switch the signal from MEC via interneurons, but there are no data on this possibility. Since MEC neurons discharge at the ascending phase of the theta rhythm in the CA1 pyramidal layer, the most likely groups of neurons that discharge at this time and inhibit apical dendrites are ivy and neurogliaform interneurons. However, these IPSPs on pyramidal neurons from the activation of these interneurons have very long characteristic times from 11 to 50 ms, which is significantly more than for the period of the middle gamma rhythm (Price et al., 2005; Krook-Magnuson et al., 2011).

The fast gamma rhythm in our model is provided by the action potentials of a large number of pyramidal neurons since the maximum excitation is observed at the minimum of the theta rhythm. Because of the overlap of excitatory inputs, a small group of pyramidal neurons gives synchronous action potentials, which are reflected in the field potential.

### Future research directions

We believe that the theoretical models proposed in neuroscience should follow the principle of Occam’s razor. Models should aim to describe in the most detail the properties of an object or phenomenon observed experimentally, while theoretical models should be as simple as possible. To date, in theoretical neuroscience, simple models have been proposed for some relatively simple experimental phenomena. For example, approximately six theoretical models have been proposed to explain the effect of phase precession of place cells (Burgess and O’Keefe, 2011). We believe that the problem of constructing models of complex phenomena can be solved by sequentially combining simple models since a balance can be maintained between the complexity of described experimental phenomena and the complexity of a model. We have tried to do in this article to describe the rhythms in the CA1 field of the hippocampus. We believe that the generalization of the proposed model to the other regions of the hippocampal formation will be a promising continuation. The dentate gyrus, field CA3, and subiculum are similar to the field CA1 by a set of populations of neurons, their connections, as well as the properties of the rhythms (Mizuseki et al., 2009; Carr et al., 2012; Buzsáki, 2015; White et al., 2020). The model of generating rhythms in the neural network of the full hippocampus, taking into account the characteristics of different regions, will have great predictive and descriptive power.

Another promising direction for the development of the proposed ideas is to combine a rhythm model with models of place cells, time cells, and grid cells. As with rhythms, many models have been proposed to explain the individual effects associated with place cells (Burgess and O’Keefe, 2011). The problem with most of these models is their small relation to rhythmic processes, but there is a lot of experimental evidence of an indissoluble connection between cognitive processes and rhythms (Vinogradova, 1995; Buzsáki and Moser, 2013; Buzsáki, 2015; Colgin, 2016). Integrating a detailed model of rhythms with models explaining remapping, pattern separation, pattern completion, and other correlates of cognitive functions at the neural level will be fundamental.

## Synthesis

Reviewing Editor: Alexey Semyanov, Shemyakin-Ovchinnikov Institute of Bioorganic Chemistry Decisions are customarily a result of the Reviewing Editor and the peer reviewers coming together and discussing their recommendations until a consensus is reached. When revisions are invited, a fact-based synthesis statement explaining their decision and outlining what is needed to prepare a revision will be listed below. The following reviewer(s) agreed to reveal their identity: Alexey Brazhe.

Two reviewers have now seen the manuscript. They both found it of some interest to specialists. However, reviewer #2 was unable to run the script and reproduce the figures. I believe this is the most critical point, and the paper can be further considered if the issue is resolved. The reviewers have also made several comments (see below) that need to be addressed.

Reviewer #1

This is a rather unusual work, containing a very complex mathematical model of a very specific object. While the results are undoubtedly useful to specialists, I cannot say that the manuscript makes progress in the field of neuroscience in general.

This is undoubtedly solid work aimed at a better understanding of the mechanisms of rhythm formation in a specific area of the hippocampus. The proposed mathematical model is very complex, and for this reason, it is unrealistic to evaluate in detail each of its elements. In general, the used model approaches are not new and therefore can be trusted.

The specific results obtained can be truly appreciated by those who are directly involved in the study of the structure and function of the hippocampus. For this reason, I find the work of limited interest for a wide group of readers.

As a specialist in modeling, but not in the study of the hippocampus, I ask the authors to further comment on the question of the unambiguity of their model. Simply put, how possible is it to find other combinations of parameters and a different structure of the model that would provide the same degree of agreement with the experimental data?

Reviewer #2

The manuscript provides a rather detailed model of hippocampal CA1 neuronal network, which, if driven by rhythmically modulated synthetic input from external connections (CA3, medial septum, entorhinal cortex), is capable of generating characteristic hippocampal LFP rhythms in theta and gamma bands. The model includes pyramidal cells as well as several types of interneurons.

The model doesn’t account for local rhythm generation mechanisms, e.g. entrainment or synchronization of local intrinsic oscillators in CA1. In essence, this model envisions and builds a complex and detailed --- yet passive --- nonlinear filter of rhythmic input from other brain structures and other hippocampal areas. Some mechanisms, such as NMDA receptors or short-term presynaptic plasticity are not regarded from the model.

The propose model puts together several models of individual neuron types from literature, and can serve as a workbench for further development and in silico hypothesis testing, regarding the mechanisms of CA1 field rhythm formation and modulation. In this respect, this work is sound and is a valuable contribution to comp. neuroscience field. Some details of the proposed model are not provided in the body or supplemental file though, and be only found in the code. The code leaves to wish for better documentation.

The Extended data includes the code accompanying code. Some of the code seems to run, but there is no README file or documentation as of package dependencies, setting up file paths, etc.

Anyone seeking to run the model code has to find out which Python modules to install via trial and error. An anaconda environment.yaml or a Docker image, not to say about a basic README file with dependencies and required steps would help to make the code more reproducible.

Some of the files in `test_scripts` directory fail with NEURON error, but others seem to be able to run. I was unable to run the whole simulation to reproduce the Figures from the manuscript.

Running `python base_model.py` fails with:

```

Traceback (most recent call last):

File “base_model.py", line 25, in <module>

run_simulation(basic_params)

File “./CA1_rhythms_model/simulation_parallel.py", line 381, in run_simulation

le = LfpElectrode(x=el_x[idx_el], y=el_y[idx_el], z=el_z[idx_el], sampling_period=h.dt, TypeError: __init__() got an unexpected keyword argument ’sec_list’

```

Apparently, some specific version of `LFPsimpy` has to be used, as the documentation for `LfpElectrode` class doesn’t allow for the `sec_list` parameter.

Model description and results

-------------------------------

- Some details of the model are missing from the body or companion technical file. For example, the numbers of neurons of each type can be found only in the accompanying source code.

- It is not readily clear from model description if only pyramidal neurons were assigned any spatial coordinate, and how were gap junction contacts assigned between PV basket cells and neurogliaform cells.

- It is interesting, to what extent the present results of the study are sensitive to inclusion of NMDA receptors and short-time presynaptic plasticity into the model.

- It appears that Figure 6 is never referred to directly in the text, but it should be instructive in understanding the roles of difference cell types in different phases of CA1 theta rhythm. Likewise, multiple panels in Figure 5 are not described or discussed in the text.

- A constant high-rate activity of axo-axonic cells catches the eye in Figures 3-4. Is it something to be expected for these cells? It seems this deserves some comment in the text.

- Why are there some sharp drops in LFP at 2000 and ∼2600 ms in Figs 3--4?

Accompanying code

--------------------------

I couldn’t run and test the provided source code (see comments above). I would suggest adding some kind of README file listing the steps required to reproduce the results from the paper along with the list of dependency packages and their respective versions. A Docker image or anaconda environment.yaml or a pip dependencies list are preferable. More detailed and self-explanatory Jupyter notebook files for simulations should also be helpful.

Text: typos, wording, etc:

---------------------------

- page 2, line 54: what is a “tought” ?

- page 2, line 60: seems to be an incomplete sentence

- page 2, line 79: describes -> describe

- page 3, line 98: “neurogliaform” (insert missing “l”)

- page 3, line 117: “weights ... were set using the Gaussian function”. It is arguably better to (i) formulate it as “weights ... were drawn from a Gaussian distribution” and (ii) provide reference values for the center and s.d. of the used distribution.

- page 3, line 119: the phrase “... center for the pyramidal neurons was shifted by 500 ms to the beginning of the simulation” is unclear. First, should weights be in milliseconds? Second, it is hard to guess, what the phrase should mean.

- page 4, line 131: “... produced by *a* current*s*"

- page 4, lines 151-152: “connections ... are large”. Should it be “... weights of connections ... are large”?

- page 4, line 157: “neurons are discharged” should be either “neurons are discharging” or “neurons discharge”.

- page 9, line 333: “smedial septum” -> “medial septum"

- page 9, line 394: “density input” -> “dense input"

## Author Response

We thank the reviewers for their numerous comments. We have corrected the article and hope that it has become clear. We marked all corrections in the text of the article with green color.

Synthesis of Reviews:

Question:

Computational Neuroscience Model Code Accessibility Comments for Author (Required):

There is no documentation on package dependencies, setting up file paths, etc.

Answer:

We added a description of files, package dependencies, paths to the README.md file in the repository on Github.

Question:

Significance Statement Comments for Author (Required):

The significance statement should outline why this work has significance: the importance of the question, the importance of the results. Currently is a set of bullet points with major findings. The author should look at significance statements of previously published papers.

Answer:

We have corrected significance statements.

Question:

Comments on the Visual Abstract for Author (Required)

Answer:

We have added the Visual Abstract.

Question:

Synthesis Statement for Author (Required):

Two reviewers have now seen the manuscript. They both found it of some interest to specialists. However, reviewer #2 was unable to run the script and reproduce the figures. I believe this is the most critical point, and the paper can be further considered if the issue is resolved. The reviewers have also made several comments (see below) that need to be addressed.

Answer:

We have corrected the code description and provided detailed instructions for running simulations. We also removed the test scripts from the master branch, leaving only the necessary files.

Reviewer #1

Question:

As a specialist in modeling, but not in the study of the hippocampus, I ask the authors to further comment on the question of the unambiguity of their model. Simply put, how possible is it to find other combinations of parameters and a different structure of the model that would provide the same degree of agreement with the experimental data?

Answer:

Another combination of parameters most likely exists, but it is a very difficult task to find it. If we take other initial conditions and another optimization method, then perhaps the result will be better. However, it is impossible to iterate over all the variants of the initial conditions. We chose a reasonable strategy for determining the initial conditions and obtained results that satisfy the provisions listed in the introduction.

We have noted this aspect in the article in the section “General notes”.

Reviewer #2

Question:

Some details of the proposed model are not provided in the body or supplemental file though, and be only found in the code. The code leaves to wish for better documentation.

Answer:

We have fixed the documentation of the code.

We added data with the number of neurons to the article.

We have added a table with the value of external currents to the supplement.

Question:

Apparently, some specific version of `LFPsimpy` has to be used, as the documentation for `LfpElectrode` class doesn’t allow for the `sec_list` parameter.

Answer:

We have modified the source code of LFPsimpy so that it was possible to simulate the contribution of only given sections (parameter sec_list), in our case only sections of pyramidal neurons. We modeled LFP only from pyramidal neurons. Our LFPsimpy branch is posted here https://github.com/ivanmysin/LFPsimpy.

Question:

Some details of the model are missing from the body or companion technical file. For example, the numbers of neurons of each type can be found only in the accompanying source code.

Answer:

The article added data on the number of neurons to the article.

Question:

It is not readily clear from model description if only pyramidal neurons were assigned any spatial coordinate, and how were gap junction contacts assigned between PV basket cells and neurogliaform cells.

Answer:

Pairs of PV and neurogliaform neurons for the gap junctions were randomly selected in accordance with their density. The parameters of the gap junctions between the PV neurons did not depend on the cell coordinates.

We have noted it in section Methods.

Question:

It is interesting, to what extent the present results of the study are sensitive to inclusion of NMDA receptors and short-time presynaptic plasticity into the model.

Answer:

This is a very interesting and difficult question. However, the answer to it requires a separate study. In the article, we have already justified the lack of NMDA receptors by the fact that there is evidence that their blockade does not change the power of the theta rhythm. Data on the role of NMDA receptors in other rhythms are also poor.

Short-term plasticity affects the patterns of neuronal discharges, provided that the input parameters change over time. The period of the slowest rhythm in our model, the theta rhythm, is 140 ms, which corresponds to the characteristic times of short-term plasticity in tens and hundreds of milliseconds (Fioravante and Regehr 2011). We consider the flow of incoming firing patterns with constant characteristics. In this case, the network will come to equilibrium and the weights of the synapses stop changing. We can say that we have considered the situation of an established equilibrium.

Question:

It appears that Figure 6 is never referred to directly in the text, but it should be instructive in understanding the roles of difference cell types in different phases of CA1 theta rhythm. Likewise, multiple panels in Figure 5 are not described or discussed in the text.

Answer:

We have added a discussion of figure 6 and the panels of figure 5 to the section Model of theta rhythm.

Question:

A constant high-rate activity of axo-axonic cells catches the eye in Figures 3-4. Is it something to be expected for these cells? It seems this deserves some comment in the text.

Answer:

There are several problems in our model. In the article, we noted a weak phase precession and a bimodal distribution of the spikes of pyramidal neurons along with the theta rhythm phase. The high-frequency activity of axo-axonal neurons is another problem that we could not solve within the framework of assumptions about the structure of the CA1 field network. We did not consider this problem important, since there are not enough axo-axonal neurons in the model - 60 units. We believe that with an increase in the scale of the model and a corresponding increase in the number of axo-axonal neurons, it is possible to reduce their firing rate by increasing the density of connections from them. We did not check this, because, with an increase in the number of axo-axonal cells, an increase in other types of neurons will be required, which will increase the necessary computing resources. Now the full model is considered for about 24 hours. An increase in the counting time will make it more difficult to solve the optimization problem.

We have noted it in the section Model of theta rhythm.

Question:

Accompanying code

--------------------------

I couldn’t run and test the provided source code (see comments above). I would suggest adding some kind of README file listing the steps required to reproduce the results from the paper along with the list of dependency packages and their respective versions. A Docker image or anaconda environment.yaml or a pip dependencies list are preferable. More detailed and self-explanatory Jupyter notebook files for simulations should also be helpful.

Answer:

We have written a README file with instructions for installing and running the model, listed all the dependencies, and specified the role of each file in the repository.

Question:

Why are there some sharp drops in LFP at 2000 and ∼2600 ms in Figs 3--4?

Answer:

For a long time, we could not determine the source of the sharp drops in LFP. They were caused by the error in the LFP simulation in one thread. We have fixed this bug. The error did not affect the results and conclusions of our article. We have corrected all the figures.

Question:

Text: typos, wording, etc:

---------------------------

- page 2, line 54: what is a “tought” ?

- page 2, line 60: seems to be an incomplete sentence

- page 2, line 79: describes -> describe

- page 3, line 98: “neurogliaform” (insert missing “l”)

- page 3, line 117: “weights ... were set using the Gaussian function”. It is arguably better to (i) formulate it as “weights ... were drawn from a Gaussian distribution” and (ii) provide reference values for the center and s.d. of the used distribution.

- page 3, line 119: the phrase “... center for the pyramidal neurons was shifted by 500 ms to the beginning of the simulation” is unclear. First, should weights be in milliseconds? Second, it is hard to guess, what the phrase should mean.

- page 4, line 131: “... produced by *a* current*s*"

- page 4, lines 151-152: “connections ... are large”. Should it be “... weights of connections ... are large”?

- page 4, line 157: “neurons are discharged” should be either “neurons are discharging” or “neurons discharge”.

- page 9, line 333: “smedial septum” -> “medial septum"

- page 9, line 394: “density input” -> “dense input"

Answer:

We thank the reviewer for these comments. We have fixed the errors.
