# Supplementary figures and images for: A Model of the CA1 Field Rhythms
Source: eNeuro. 2021 Nov 8;8(6):ENEURO.0192-21.2021. doi: 10.1523/ENEURO.0192-21.2021 (PMC8577063; doi:10.1523/ENEURO.0192-21.2021)

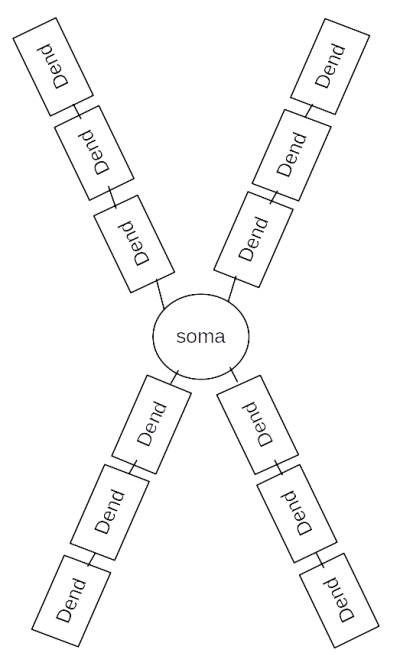

Supplement: Extended Data 1 — Latex supplement. Download Extended Data 1, ZIP file. [file enu-eN-NWR-0192-21-s03.zip › figures/bis_structure.png]

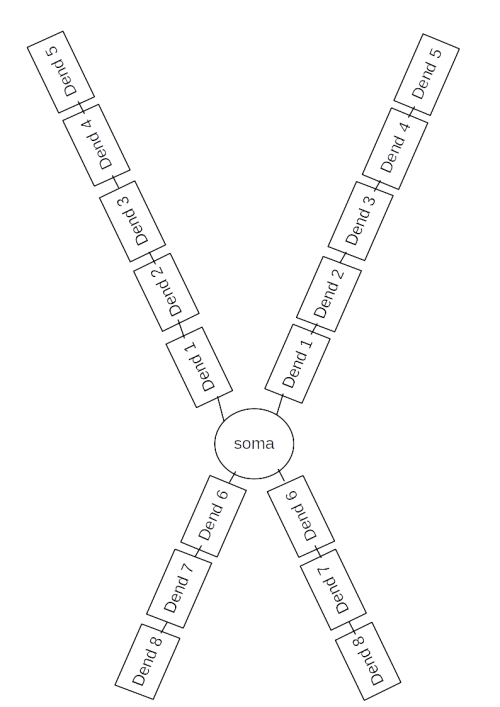

Supplement: Extended Data 1 — Latex supplement. Download Extended Data 1, ZIP file. [file enu-eN-NWR-0192-21-s03.zip › figures/interneuron_structures.png]

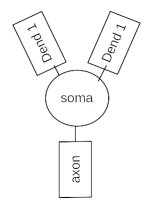

Supplement: Extended Data 1 — Latex supplement. Download Extended Data 1, ZIP file. [file enu-eN-NWR-0192-21-s03.zip › figures/olm_structure.png]

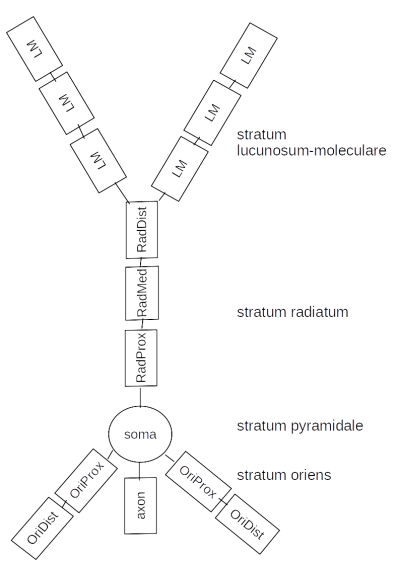

Supplement: Extended Data 1 — Latex supplement. Download Extended Data 1, ZIP file. [file enu-eN-NWR-0192-21-s03.zip › figures/pyramide_structures.png]
